# Endovascular Treatment of Tiny Aneurysms With Low-Profile Visualized Intraluminal Support Devices Using a “Compressed” Stent Technique

**DOI:** 10.3389/fneur.2020.610126

**Published:** 2020-12-18

**Authors:** Yangyang Zhou, Qichen Peng, Xinzhi Wu, Yisen Zhang, Jian Liu, Xinjian Yang, Shiqing Mu

**Affiliations:** Department of Interventional Neuroradiology, Beijing Neurosurgical Institute, Beijing Tiantan Hospital, Capital Medical University, Beijing, China

**Keywords:** tiny aneurysm, endovascular treatment, coils, compression technique, LVIS stent

## Abstract

**Objective:** To investigate the safety and efficacy of low-profile visualized intraluminal support (LVIS) stent-assisted coiling of intracranial tiny aneurysms using a “compressed” stent technique.

**Methods:** We retrospectively analyzed patients with tiny aneurysms treated in our hospital with LVIS devices using a compressed stent technique. We analyzed patients' imaging outcomes, clinical outcomes, and complications.

**Results:** Forty-two tiny aneurysms in 42 patients were included in this study cohort; 8 patients presented with subarachnoid hemorrhage at admission. The immediate postoperative complete embolization rate was 76.2% (32/42). After an average of 8.5 months of imaging follow-up, the complete embolization rate was 90.5% (38/42), and no aneurysm recanalization occurred. After an average of 24.4 months of clinical follow-up, 95.2% (40/42) of the patients achieved favorable clinical outcomes (modified Rankin scale = 0/1). Operation-related complications occurred in two patients (4.8%); one intraoperative acute thrombosis, and one significant unilateral decreased vision during the postoperative follow-up.

**Conclusion:** LVIS stent-assisted coiling of intracranial tiny aneurysms using a compressed stent technique is safe and effective. Combined stent compression technology is beneficial to maximize the complete embolization of aneurysms and reduce aneurysm recanalization. This study expands the clinical applicability of LVIS stents.

## Introduction

**Figure 1:**
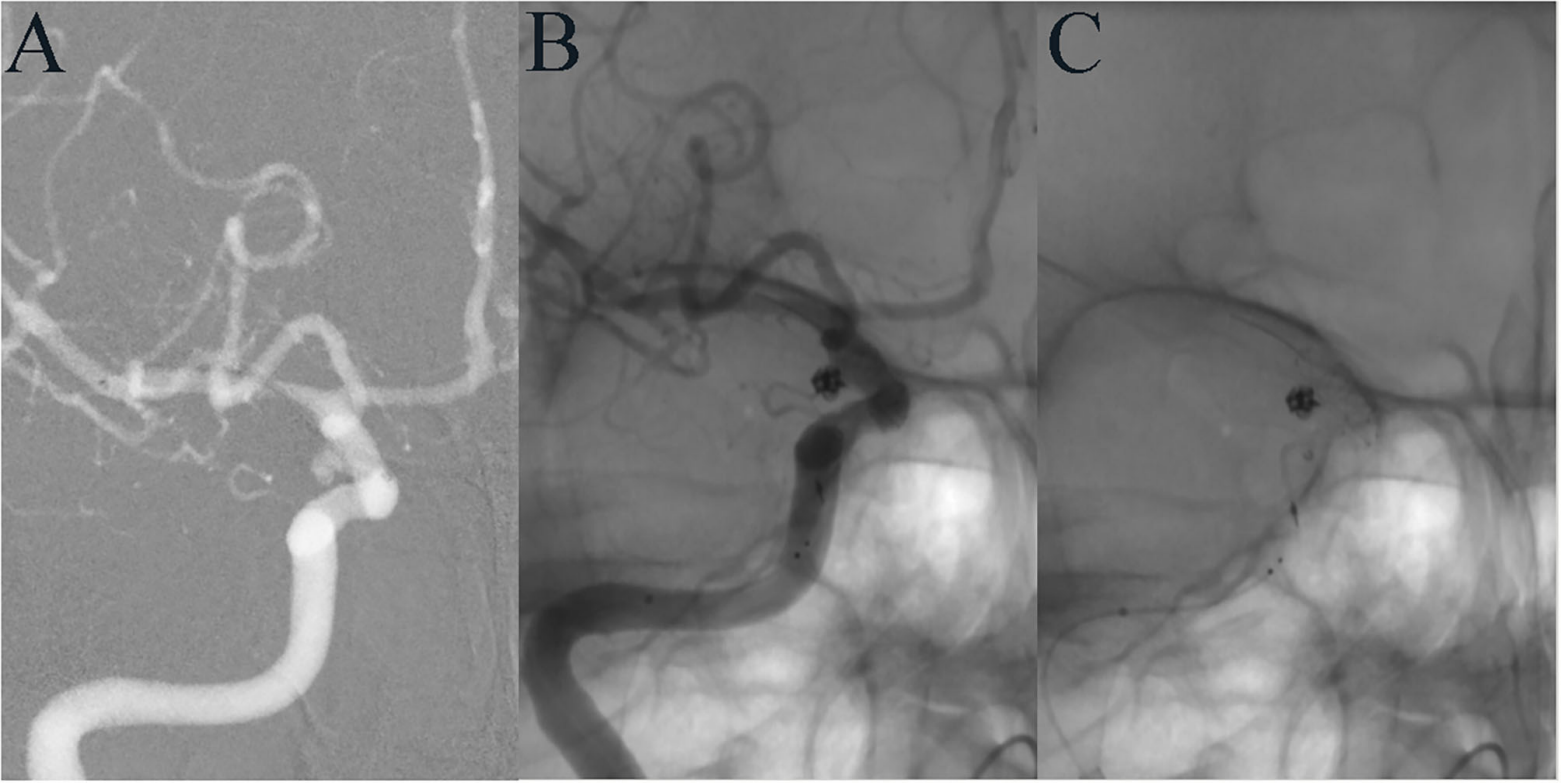
A 35-year-old female developed mild dizziness. DSA showed a tiny aneurysm in the communicating segment of the ICA **(A)**. LVIS-assisted coil embolization was performed using the compressed stent technique, and the aneurysm was completely embolized immediately after operation **(B)**. The LVIS stent at the aneurysmal neck was densely compressed **(C)**.

Intracranial tiny aneurysm refers to aneurysms with a largest diameter of ≤3 mm (1–3). Tiny aneurysms are being increasingly diagnosed with rapid developments in imaging techniques. Compared with aneurysms of other sizes, tiny aneurysms often present with thin aneurysmal walls (4, 5), and neurosurgical clipping is challenging. When the diameter is <3 mm, which equals the width of the aneurysmal clip blade, the clip will not effectively close the aneurysmal neck and may cause avulsion of the aneurysmal neck or parent artery (6). Similarly, interventional embolization aneurysmal therapy also involves difficulties. During embolization, it is difficult to insert the microcatheter into the narrow aneurysm cavity, and the coil cannot be successfully bent and rotated into the aneurysm cavity. More serious is the possibility that the microcatheter or coil may pierce the aneurysm wall leading to subarachnoid hemorrhage (SAH) (2). Therefore, the very question of whether tiny aneurysms should be treated is controversial. Low-profile visualized intraluminal support (LVIS) devices are self-expanding nickel titanium (nitinol), single-wire braid, closed-cell microstents. Compared with traditional intracranial stents, such as the Enterprise, Neuroform, and Solitaire, LVIS devices have a smaller cell structure (1.0 × 0.3 mm) and higher metal coverage (up to 23%), which can effectively prevent small coils from protruding into the parent artery. LVIS devices also function in remodeling the aneurysmal neck and in flow diversion (7). When placing the LVIS device, using a compressed stent technique at the aneurysm neck can effectively increase the metal coverage and achieve dense embolization of the aneurysm cavity and reduce postoperative aneurysm recanalization (Figure 1). We studied patients in a single center to analyze the safety and efficacy of LVIS devices for treating tiny aneurysms using a compressed stent technique.

## Methods

### Patient Selection

We retrospectively analyzed patients who were diagnosed with a tiny aneurysm by digital subtraction angiography (DSA) and who were treated with an LVIS device combined using a compressed stent technique in our hospital from July 2016 to December 2018. All aneurysms measured no more than 3 mm in size and included ruptured and unruptured aneurysms. The stent release process and medical records were reviewed to judge whether the compressed stent technique was used. Patients who did not undergo postoperative imaging follow-up were excluded. This study was approved by the Institutional Review Board of Beijing Tiantan Hospital and all patients agreed to participate and signed informed consent forms.

### Data Collection and Follow-Up

Reviewing patients' medical records, we collected demographic information, such as sex, age, chief complaint, lifestyle habits, and underlying disease. We examined the imaging data to obtain the anatomical characteristics of the tiny aneurysms, such as size, shape, and location. Follow-up imaging and telephone follow-ups were performed for each patient to analyze the aneurysm embolization outcomes and patients' clinical outcomes, respectively. Postoperative follow-up imaging constituted mainly DSA and computed tomographic angiography (CTA). Embolization classification of each aneurysm was done in accordance with the O'Kelly–Marotta grading scale (D: complete occlusion; C: trace filling; B: entry remnant; A: aneurysm filling), including for immediate postoperative imaging and postoperative follow-up imaging. We followed-up each patient by telephone to determine how their initial symptoms changed and whether there were postoperative complications. Patients' final clinical outcomes were classified in accordance with the modified Rankin scale (mRS), with an mRS score of 0–1 indicating a favorable outcome.

### Endovascular Procedure

All operations were performed under general anesthesia. The modified Seldinger technique was used to perform unilateral femoral artery puncture and to implant the arterial sheath. The parent artery was first performed with conventional three-dimensional rotatory angiography. After selecting the appropriate working position, a Headway-21 microcatheter (MicroVention, CA, USA) was implanted into the parent artery under the guidance of the microguidewire. An Echelon-10 microcatheter (Medtronic, MN, USA) was then placed into the aneurysm cavity. The LVIS device (MicroVention, CA, USA) was then delivered to the distal end of the parent artery. The first coil was partially filled to form the first ring, and the stent was then completely released. With the distal end of the stent fully adherent to the wall, we fixed the stent microcatheter to maintain tension while compressing the stent, and after maximum stent shortening with compression, the stent was slowly released. For aneurysms at bifurcations or aneurysms with important branches near the neck, the stent was released in a “lantern-like” shape after compression, meaning that the diameter of the stent was wider at the aneurysm neck. C-arm flat-detector CT was performed to observe stent apposition. Then, with the jailing technique, the first coil was filled completely, and several coils were filled in turn until the aneurysm was completely embolized and the operation was completed. All interventional procedures were performed by neurointerventionists with more than 10 years of embolization experience in our hospital.

### Drug Administration

Patients with unruptured aneurysms took oral dual antiplatelet drugs for at least 5 days preoperatively, namely aspirin 100 mg per day and clopidogrel 75 mg per day. A bolus of 3,000 IU of heparin was administered intravenously after femoral arterial sheath placement. For ruptured aneurysms, if the patient was awake, 300 mg clopidogrel and 300 mg aspirin were administered orally before the procedure, without the single bolus of intravenous heparin. If the patient was unconscious and unable to take antiplatelet drugs orally, a single dose of 6 ml tirofiban (50 μg/ mL) was injected intravenously after stent release, and tirofiban was administered continuously intravenously at 6 ml/h throughout the operation. All patients received local heparinization throughout the operation as a continuous heparin saline infusion via the guiding catheter at ~1,000 IU per hour. After discharge, patients continued to take aspirin 100 mg/day for 1 year, and clopidogrel 75 mg/day for 3 months.

## Results

### Patients' and Aneurysm Characteristics

**Figure 2:**
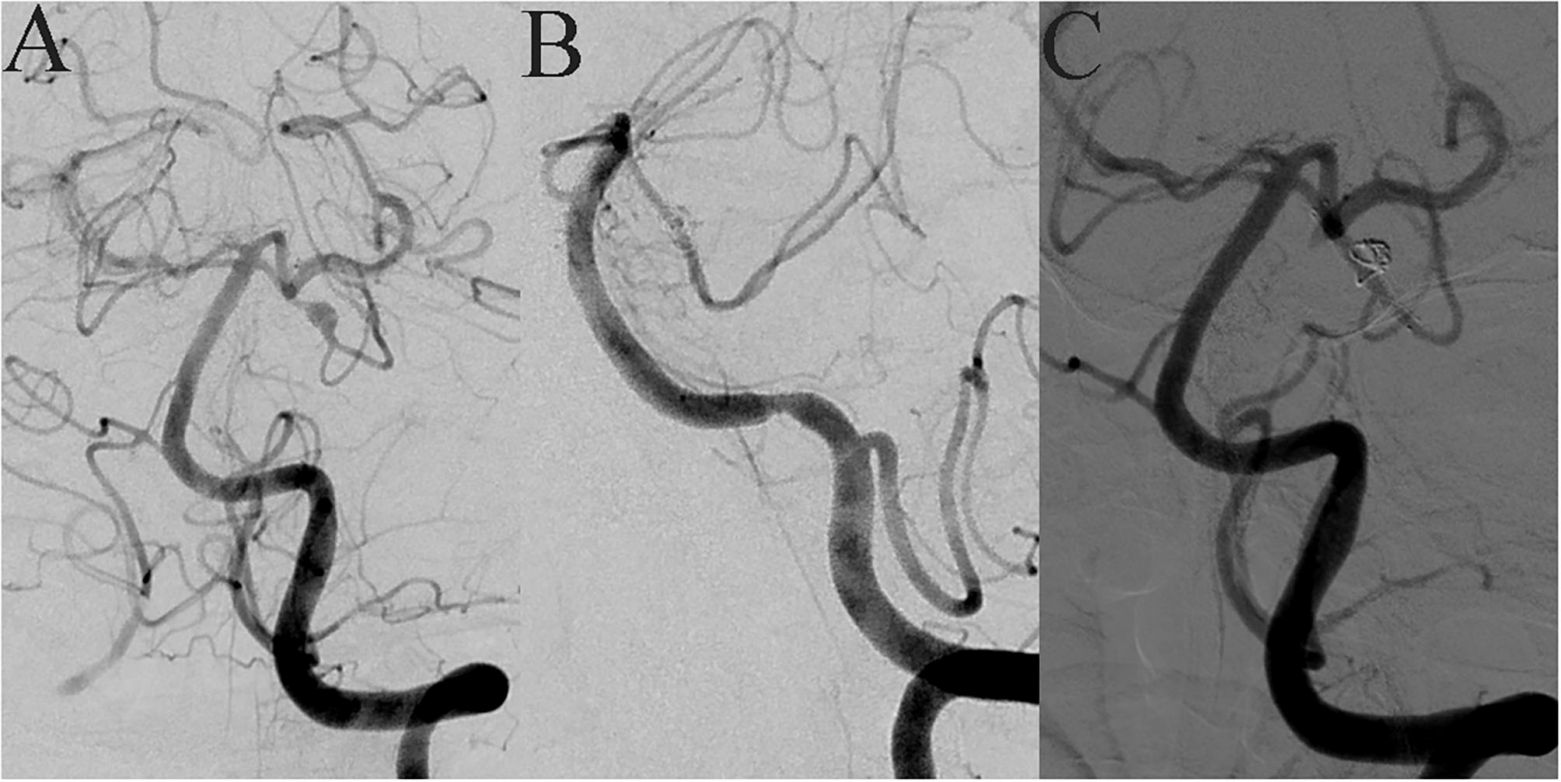
A 42-year-old female suddenly developed severe headache with nausea and vomiting when she was 7 months' pregnant. MRA showed an intracranial tiny aneurysm, and she underwent conservative treatment. Seventeen months later, she came to our hospital for interventional embolization. DSA showed a superior cerebellar artery tiny dissecting aneurysm **(A)**. LVIS-assisted coil embolization was performed, and almost complete embolization of the aneurysm was achieved immediately after operation **(B)**. The LVIS device retained a good shape after compression release **(C)**.

Forty-two tiny aneurysms in 42 patients were included in this study. The mean patient age was 52.1 ± 10.9 years (range, 28–68 years), and 15 patients were male. Eight patients presented with SAH at admission, and all were Hunt–Hess grade < II. All patients with unruptured aneurysms had mRS scores <2 at admission. Most of the aneurysms were located in the anterior circulation, with the internal carotid artery accounting for 83% (35/42). Only one aneurysm was a fusiform aneurysm (Figure 2); all others were saccular aneurysms. All aneurysms had a maximum diameter of no more than 3 mm and an average diameter of 2.4 ± 0.3 mm (range, 1.8–3.0 mm). Detailed patient and aneurysm characteristics are shown in Table 1.

**Table 1:** Patients' baseline characteristics.

**Characteristic**	**Number**
Number of patients	42
Number of aneurysms treated	42
SAH, *n* (%)	8 (19.0)
Male sex, *n* (%)	15 (35.7)
Age (years), mean ± SD	52.1 ± 10.9
Smoking, *n* (%)	6 (14.3)
Alcohol use, *n* (%)	4 (9.5)
Hypertension, *n* (%)	9 (21.4)
Hyperlipidemia, *n* (%)	5 (11.9)
**Pretreatment mRS**, ***n*** **(%)**	
0	14 (33.3)
1	26 (61.9)
2	2 (4.8)
**Aneurysm location**, ***n*** **(%)**	
ICA	35 (83.2)
AcoA	2 (4.8)
PcoA	2 (4.8)
PICA	2 (4.8)
SCA	1 (2.4)
**Morphology**	
Saccular and wide neck	37 (88.1)
Saccular and narrow neck	4 (9.5)
Fusiform	1 (2.4)
Size, mm, mean ± SD	2.4 ± 0.3

*Data are presented as n, n (%), or mean ± standard deviation. SAH, subarachnoid hemorrhage; ICA, internal carotid artery; AcoA, anterior communicating artery; PcoA, posterior communicating artery; PICA, posterior inferior cerebellar artery; SCA, superior cerebellar artery*.

### Operation Outcomes and Follow-Up

Forty-two LVIS stents were used for 42 tiny aneurysms, and all stents were implanted successfully. No intraoperative aneurysm rupture occurred. Thirty-two (76.2%) tiny aneurysms were completely embolized immediately after interventional embolization, and 7 (16.7%) tiny aneurysms showed trace filling. All patients underwent imaging follow-up at least once. After an average of 8.5 months of imaging follow-up, 38 (90.5%) aneurysms showed complete embolization, three (7.1%) aneurysms showed trace filling, and only one (2.4%) aneurysm showed an entry remnant. No aneurysm recanalization occurred. Postoperative clinical follow-up was conducted for an average of 24.4 months. At the final follow-up, 24 patients showed no symptoms (mRS = 0), and 16 patients (mRS = 1) showed mild symptoms, such as occasional headache and dizziness. The rate of favorable clinical outcomes reached 95.2% (40/42). The treatment outcome details are shown in Table 2.

**Table 2:** Treatment outcomes and follow-up.

**Results**	**Number**
**Operative time (minutes)**	
Mean ± SD	91.0 ± 31.2
**Postoperation immediate aneurysm occlusion, OKM**, ***n*** **(%)**	
D	32 (76.2)
C	7 (16.7)
B	3 (7.1)
D + C	39 (92.9)
**Aneurysm occlusion at last follow-up, OKM**, ***n*** **(%)**	
D	38 (90.5)
C	3 (7.1)
B	1 (2.4)
D + C	41 (97.6)
Follow-up time (months)	8.5 ± 3.5
**mRS at last follow-up**, ***n*** **(%)**	
0	24 (57.1)
1	16 (38.1)
2	1 (2.4)
5	1 (2.4)
Follow-up time (months)	24.4 ± 9.2
**Operation complications**, ***n*** **(%)**	
Coil prolapse	1 (2.4)^*^
Thromboembolic events	1 (2.4)^*^
Neurologic deficits	1 (2.4)
Total	2 (4.8)

* *Two complications occurred in one patient. Data are presented as n, n (%), or mean ± standard deviation; OKM, O'Kelly–Marotta grading scale; mRS, modified Rankin scale*.

**Figure 3:**
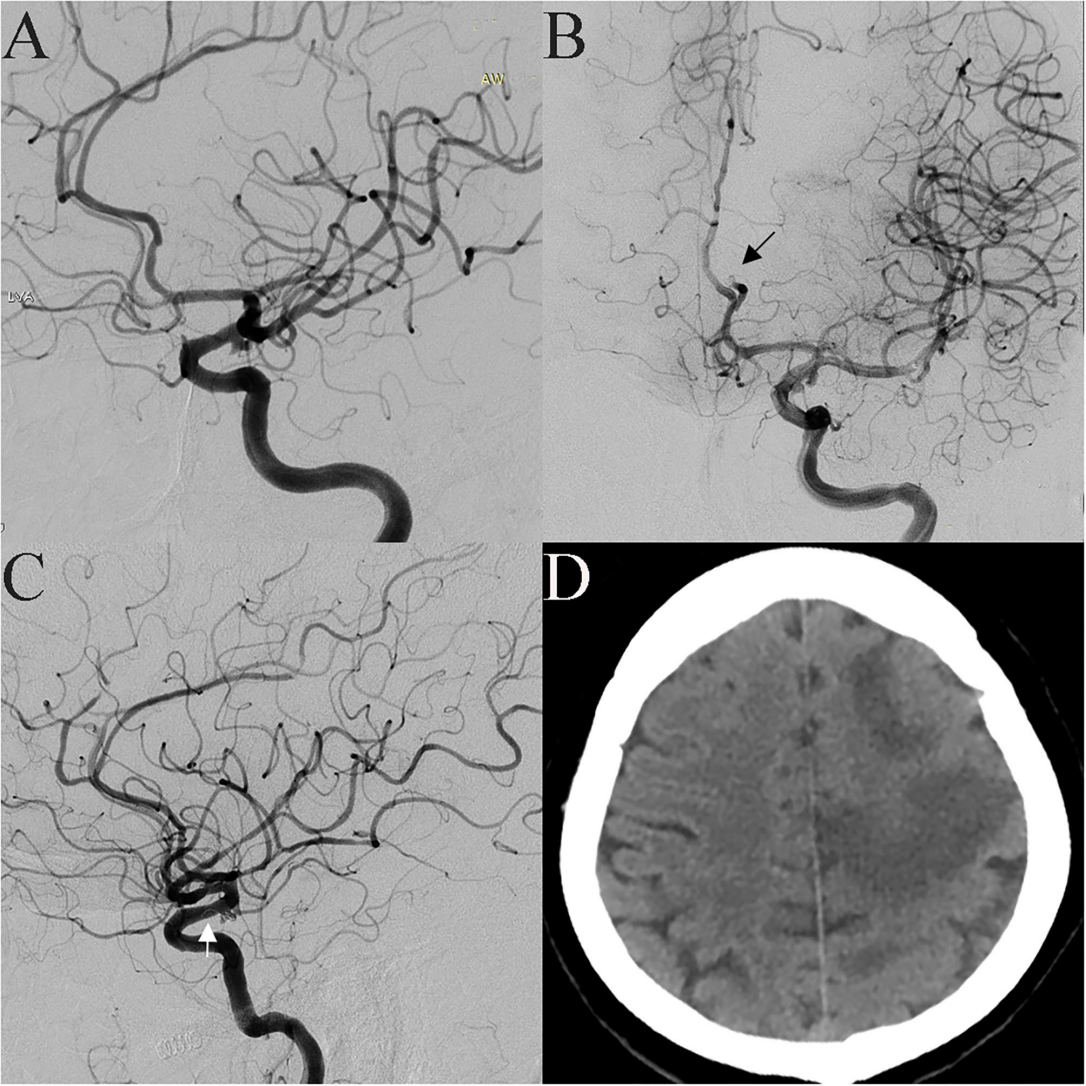
A 53-year-old female was incidentally found have a tiny aneurysm in the communicating segment of the ICA **(A)**. Pericallosal artery occlusion was found when the microcatheter was advanced to the aneurysm cavity, and a thrombus was seen (**B**, black arrow). After intravascular thrombolysis, patency in the artery was partly restored. The coil prolapsed into the parent artery when the first coil was placed; therefore, an LVIS stent was implanted (**C**, white arrow). Postoperative CT scan showing a large infarction in the frontal parietal lobe **(D)**.

**Figure 4:**
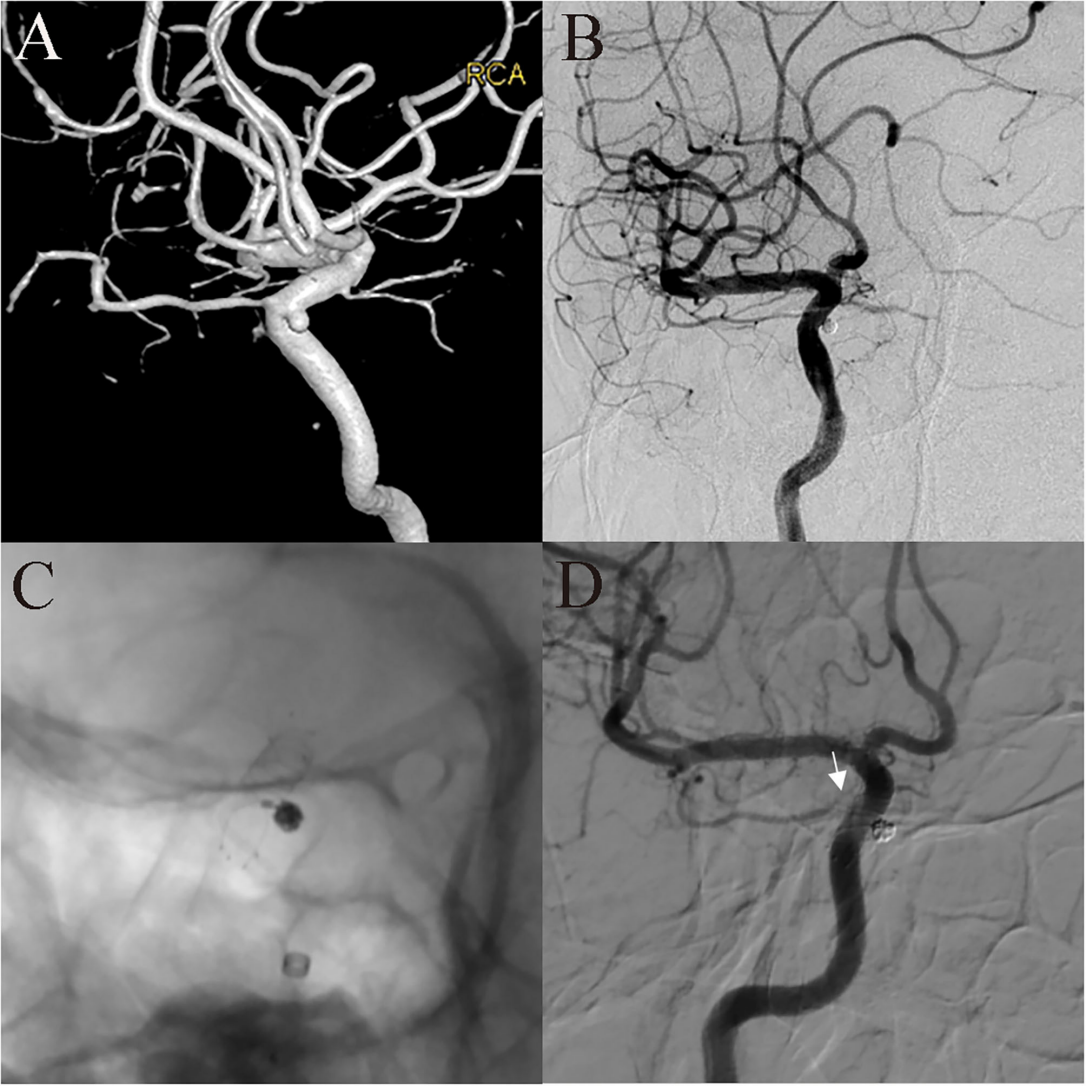
A 52-year-old female patient with intermittent dizziness for 1 year was found to have a tiny aneurysm. DSA showed a small aneurysm in the ophthalmic segment of the ICA **(A)**, and LVIS-assisted coil embolization was performed using the compressed stent technique. Immediately after embolization, the aneurysm was completely embolized **(B)**, and the ophthalmic artery was patent. The LVIS stent was compressed at the aneurysm neck **(C)**. The 14-month follow-up images showed no recurrence of the aneurysm, but the initial segment of the ophthalmic artery was narrowed (**D**, white arrow).

One patient was originally scheduled for simple coil embolization. After placing the first coil, the coil prolapsed into the parent artery by ~0.5 cm; therefore, we chose to implant an LVIS device to compress the coil and stop it from falling into the parent artery completely. This patient was also the only patient with intraoperative complications. After general anesthesia, the callosomarginal artery was found to be occluded due to acute thrombosis during the implantation of the coil microcatheter. The patient underwent immediate endovascular thrombolysis treatment, and the callosomarginal artery was recanalized. After the operation, the patient presented with grade 2 muscle strength on one side of the body and speech difficulties. Head CT scan showed a large frontal parietal infarction, and the patient's mRS score was 5 when discharged (Figure 3). Another patient developed postoperative significant unilateral vision loss. The image of the patient 14 months after the operation showed initial stenosis of the ophthalmic artery, but the patient had not recently undergone an imaging evaluation at the time of vision loss. We considered that the LVIS stent possibly limited the ophthalmic artery blood flow (Figure 4).

## Discussion

### Treatment of Tiny Aneurysms

There is currently no fixed definition of tiny aneurysm, and different terms, such as very small aneurysm and microaneurysm, appear in different studies (1, 8). There is also no consensus regarding the treatment method, and common treatment methods include craniotomy for clipping, and interventional embolization. Chalouhi et al. reported 60 tiny aneurysm patients who underwent craniotomy for clipping, with operation complications of up to 23.3%, and 91 microaneurysms that underwent interventional embolization with operation complications of 9.8% (9). In contrast, Molyneux et al. stated that tiny aneurysms were more suitable for craniotomy clipping considering that interventional embolization was associated with difficulties in catheterizing the aneurysm, stabilizing the microcatheter, and safely deploying coils (10). The narrow aneurysm cavity and thin aneurysm wall are associated with a high intraprocedural rupture rate (11). Some studies have reported that the intraoperative rupture rate of small aneurysms was twice or even five times that of larger aneurysms (12, 13). A meta-analysis of very small (≤3 mm) intracranial aneurysms showed a higher intraprocedural rupture rate in both unruptured (5.0%) and ruptured aneurysms (10.7%), while the mortality rate due to procedural rupture was 2.4% (2). According to the International Subarachnoid Hemorrhage Test (ISHT), the 5-year rupture rate of aneurysms with a diameter <7 mm is <0.7%, while that of aneurysms located in the anterior circulation is almost 0 (14). Therefore, whether unruptured aneurysms require active intervention has aroused great controversy (15). However, according to a recent study, the rate of rupture during interventional therapy for tiny aneurysms was significantly lower, at 1% (16). With improved embolization materials, such as smaller and softer coils, steerable microcatheters, and increased embolization skill, there were no intraoperative aneurysm ruptures in any of our 42 patients. The following operation techniques may help reduce intraoperative aneurysm rupture: First, conduct reasonable modeling of the microcatheter to make it easy to enter the aneurysm cavity, and push the coil microcatheter across the aneurysmal neck and back down, to allow it to fall naturally into the arterial cavity, which can effectively prevent the microcatheter tip from acting directly on the aneurysm wall. Second, the LVIS stent combined with the compressed stent technique increases the metal coverage of the aneurysm neck and fixes the coil microcatheter for coil packing. Third, it is best to choose an ultra-soft coil and one smaller than the diameter of the aneurysm. In a word, interventional embolization for tiny aneurysms is currently considered safe and feasible, and treatment is considered necessary for ruptured aneurysms. However, for unruptured tiny aneurysms, treatment is usually necessary only under certain conditions, such as aneurysms with an irregular shape, multiple aneurysms, aneurysm growth during follow-up, a family history of SAH, or patients with severe anxiety. All treated aneurysms in our study met these operation indications.

### LVIS Stents and the Compressed Stent Technique

Compared with simple coil embolization, stent-assisted coil embolization has obvious advantages. Stent-assisted coil embolization can effectively reduce coils escaping from the aneurysm cavity, increase coil packing density, and reduce aneurysm recurrence (17, 18). However, the long-term use of dual antiplatelet drugs after stenting increases the risk of aneurysm bleeding. The LVIS device is a self-expanding nickel titanium (nitinol), single-wire braid, closed-cell microstent. These devices are an intermediate choice between conventional stents and flow diverters, and the devices have the advantages of closed-cell stents, such as strong radial support, as well as the advantages of open-cell stents such as good stent–vessel wall apposition. LVIS devises also have good blood flow diversion. The LVIS stent has two radiopaque strands (the LVIS Jr has three) along its entire length as well as four proximal and distal radiopaque markers, allowing the neurointerventionist to visualize full stent release and which also provide the possibility of adopting the compressed stent technique (19, 20). Other conventional intracranial stents have markers only at the proximal and distal tines. In addition, the metal LVIS stent coverage rate is up to 23%, which is higher than the traditional Neuroform and Enterprise stents at 11 and 9%, respectively. Liu et al. reported 54 patients with small aneurysms treated with conventional stent-assisted coil embolization, namely with the Enterprise, Neuroform, and Solitaire stents, and reported that 80% of aneurysms were eventually completely embolized (21). This rate was lower than the rate of 90.5% in our study. Lee et al. reported 312 small-sized aneurysms (<10 mm) treated with different stents, in which the aneurysm recanalization rate after LVIS stenting was 6.5%; a much lower rate than with the Enterprise (18.2%) and Neuroform stents (29.6%) (22).

Using the compression technique to release the stent requires skillful operation experience. Post-stent release or partial stent release is often performed because stent compression release limits the movement of the coil microcatheter in the aneurysmal neck. First, the distal end of the stent is attached to the vessel wall for release, then stent compression is initiated when the distance from the microcatheter tip to the aneurysm neck center is 1.2–1.5 times the diameter of the parent vessel. The microcatheter is pushed and kept in maximum tension but not bent, then the microcatheter is fixed, and the stent is slowly released, which concentrates stent distribution in the aneurysm neck. Finally, the remainder of the stent is released. This approach results in better stent compression at the aneurysmal neck. If there are branched vessels near the aneurysmal neck, adding a “lantern” release technique can avoid blocking blood flow in the branches. In this study, we treated 42 tiny aneurysms with the compressed stent technique, and achieved an immediate complete embolization rate of 76.2%. In previous studies using conventional stent release techniques, Wu et al. reported an immediate postoperative embolization rate of 46.4% and Gao et al. reported a rate of 36.4% (23, 24). After an average of 8.5 months of postoperative imaging follow-up, 90.5% of the aneurysms finally achieved complete embolization, and no aneurysms were recanalized. Meanwhile, Gao et al. reported complete and near-complete embolization rates of 81.8%, and Wu et al. reported an embolization rate of 78.1%. These results show that the compressed stent technique can effectively improve the total embolization rate of aneurysms and reduce the recanalization rate. With the compressed stent technique, the LVIS metal mesh is denser and effectively prevents the coil from escaping from the stent pores, thus reducing the rate of infarction of the parent artery or terminal vessel (19). Furthermore, the compression technique increases metal coverage by the LVIS at the aneurysm neck, which not only changes the hemodynamic characteristics of the aneurysm, but also provides a good platform for intimal growth and vascular wall repair, thereby reducing aneurysm recurrence. Finally, the higher degree of metal surface area coverage at the aneurysm neck provides a more robust flow diversion effect compared with the other available stents (25). These factors likely account for the very high levels of complete and adequate aneurysm occlusion observed in the present study.

### Operation Complications

Thrombotic events are the most common operation complications with embolization therapy of intracranial aneurysms with LVIS devices. A meta-analysis of nine studies reported an overall operation-related complication rate of 6.5% and a thrombotic event rate of 4.9% (19). In our study, one patient developed an acute intraoperative thrombus, and despite immediate intravascular thrombolysis and recanalizing the vessel, the patient unfortunately developed severe postoperative disability. Interventional embolization of the intracranial aneurysm was performed intravascularly; however, despite careful preoperative preparation and gentle intraoperative procedures, acute endovascular thrombosis still occurred. The main causes of acute thrombosis may be related to factors such as long intraoperative operation time, intraoperative endothelial damage, insufficient anticoagulation, activation of the coagulation system, and thrombogenicity of interventional embolization materials (26). Although dense mesh stents have many advantages over traditional stents, dense stents also increase the possibility of occlusion of branch arteries near aneurysms (27), especially when combined with the compression technique. Additionally, the LVIS device's high-profile mesh may block blood flow in the branch. After 24 months of imaging follow-up, an ophthalmic artery aneurysm patient in this study presented with ipsilateral significant vision loss. However, the patient had not undergone recent imaging. Combined with images taken immediately after embolization and at 14 months, we suspect that there may have been severe narrowing of the ophthalmic artery. Therefore, when there is a branch vessel near an aneurysm, “lantern-like” release should be adopted. If there is technical difficulty, the compression technique should be abandoned, and tension-free release should be adopted.

## Limitations

This study was a retrospective, single-center study. Additionally, tiny aneurysms were less common in all aneurysms and not all patients received follow-up evaluations; therefore, we were not able to obtain a large sample size. The mean imaging follow-up time for all patients was 8.5 months, which was too short to evaluate the final complete embolization rate. Most of the aneurysms in this study were located in the internal carotid artery, which differed from the distribution of most aneurysms, and this may have resulted from neurointerventionist selection. In addition, subjective manual measurement affected the accuracy of the tiny aneurysm size.

## Conclusions

LVIS-assisted coiling for intracranial tiny aneurysms combined with compressed stent release is safe and effective. Combined stent compression technology is beneficial to maximize the complete embolization of aneurysms and reduce aneurysm recanalization. This study expands the clinical applicability of LVIS stents.

## Data Availability Statement

The raw data supporting the conclusions of this article will be made available by the authors, without undue reservation.

## Ethics Statement

The studies involving human participants were reviewed and approved by Institutional Review Board of Beijing Tiantan Hospital. The patients/participants provided their written informed consent to participate in this study.

## Author Contributions

YZho collected the clinical data and wrote the manuscript. QP and XW helped collect the clinical data. YZha and JL wrote sections of the manuscript. SM and XY helped revise the manuscript. SM designed the research and handled funding and supervision. All authors read and approved the final manuscript.

## Conflict of Interest

The authors declare that the research was conducted in the absence of any commercial or financial relationships that could be construed as a potential conflict of interest.
